# Rhegmatogenous retinal detachment after a lower extremity dog bite: a case report

**DOI:** 10.1186/1757-1626-1-218

**Published:** 2008-10-07

**Authors:** Tarek Alasil, Sam Eljammal, Richard Scartozzi, Dean Eliott

**Affiliations:** 1 University of Southern California and Doheny Eye Institute, Los Angeles, California 90033, USA

## Abstract

**Introduction:**

The occurrence of endogenous endophthalmitis is well known in the settings of bacteremia or candidemia, although its association with rhegmatogenous retinal detachment remains unclear.

**Case presentation:**

We present a case of rhegmatogenous retinal detachment in a 39 year old man who presented with acute vision loss six weeks after a lower extremity dog bite. Further workup revealed oxacillin resistant staphylococcus aureus bacteremia with multiple septic emboli to various organs including bilateral loculated pleural effusion, left renal abscess, left obturator abscess, and right eye endogenous endophthalmitis.

**Conclusion:**

Our case illustrates an interesting presentation of oxacillin resistant staphylococcus aureus bacteremia after a dog bite, where endogenous endophthalmitis and rhegmatogenous retinal detachment were part of the manifestations. Early diagnosis and intervention are crucial to preserve the visual acuity.

## Introduction

Endophthalmitis is an ocular inflammation resulting from the introduction of an infectious agent into the posterior segment of the eye. During infection, irreversible damage to delicate photoreceptor cells of the retina frequently occurs. Despite aggressive therapeutic and surgical intervention, endophthalmitis generally results in partial or complete loss of vision, often within a few days of inoculation. Infectious agents generally gain access to the posterior segment of the eye either postoperatively, post-traumatic, or from hematogenous spread of bacteria from a distant anatomical site (endogenous) [1].

Endogenous endophthalmitis is relatively rare, accounting for only 2 to 8% of all endophthalmitis cases [2-4]. Populations at greatest risk include immunocompromised patients or those on immunosuppressive therapy, patients with prolonged indwelling devices, and intravenous drug abusers [5,6].

Common causes of endogenous bacterial endophthalmitis include S. aureus, B. cereus, and gram-negative organisms, including Escherichia coli, Neisseria meningitidis, and Klebsiella species [4,5,7,8]. Bacillus species are a primary bacterial cause of endogenous endophthalmitis in intravenous drug abusers and are most likely seeded from contaminated injection paraphernalia and drug solutions [2,5,6]. The most common etiological agent of all cases of endogenous endophthalmitis is the opportunistic fungus Candida albicans [4].

The occurrence of endogenous endophthalmitis is well known in the settings of bacteremia or candidemia, although its association with rhegmatogenous retinal detachment remains unclear.

## Case presentation

A 39 year old male with a history of dog bite to the left lateral thigh during a police pursuit, was bought into the emergency room from jail complaining of acute onset vision loss in the right eye, shortness of breath, and left leg pain six weeks after the dog bite.

The wound was initially treated with oral antibiotics, and mildly improved. However, it became indurated later with painful tracking to the left hip.

Social history was significant for amphetamine abuse in the past.

Upon presentation to the Emergency room, the patient was found to have tachycardia, and leukocytosis. The vital signs are summarized in table 1. The laboratory workup is summarized in table 2.

**Table 1:** Vital signs upon presentation

	Blood pressure	Heart rate	Temperature	Respiratory rate
Vital signs	121/67	129	99.9 F	20

**Table 2:** Laboratory workup upon presentation

Sodium	133
Potassium	4.2
Chloride	103
Bicarbonate	18
Blood urea nitrogen	19
Creatinine	0.7
White blood cell	18.6
Red blood cell	3.06
Hemoglobin	9.3
Hematocrit	26.7
Platelets	413
Neutrophils	90.8%
HIV Elisa test	Negative
Hepatitis C virus antibody	Negative
Hepatitis B surface antigen	Negative
Hepatitis B surface antibody	Negative
CRP	224
ESR	105

**Figure 1:**
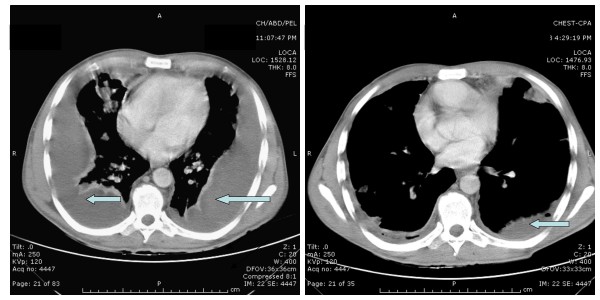
**CT scans of the chest**. Left: Computed Tomography scan of the chest upon initial presentation is showing bilateral pleural effusion and parenchymal lung disease. Right: Computed Tomography scan of the chest post treatment with intravenous antibiotics and bilateral pig tail tubes placement, is showing improvement in the bilateral pleural effusions and the parenchymal lung disease.

**Figure 2:**
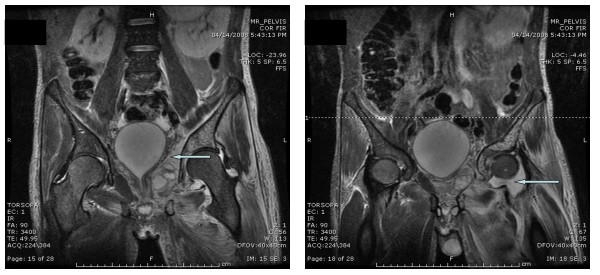
**MRI of the pelvis**. Left: Magnetic resonance imaging of the pelvis is showing extensive irregular collection (abscesses) along the left obturator internus muscle with infiltration to the surrounding left hip structures (arrow). Right: Magnetic resonance imaging of the pelvis is showing left hip joint effusion that is suggestive of septic joint (arrow).

Further workup revealed left thigh abscess and signs of sepsis. The patient was transferred to the intensive care unit. Blood cultures showed oxacillin resistant staphylococcus aureus (ORSA) bacteremia, and further evaluation revealed multiple septic emboli to various organs, causing bilateral loculated pleural effusion (Figure 1), left renal cyst abscess, left obturator abscess (Figure 2).

The patient was initiated on intravenous levofloxacin, vancomycin, and gentamycin. He underwent emergent hip incision and drainage, computed tomography guided placement of bilateral pigtail tubes to drain the pleural fluid, and a nephrostomy tube was placed to drain the left renal cystic abscess. Trans-esophageal echocardiogram showed no evidence of endocarditis. The left hip arthrocentesis and the left renal cystic fluid both grew ORSA.

Meanwhile, the ophthalmology service was consulted to evaluate the right eye acute vision loss in the setting of systemic ORSA bacteremia. Initial ophthalmological exam of the right eye showed visual acuity of 20/200 on the right and 20/25 on the left. Intraocular pressure was 10 mmHg bilaterally. Slit lamp exam of the right eye showed conjunctival injection, corneal edema, chemosis, anterior chamber cell and flare. Slit lamp exam on the left was normal. Dilated fundus exam of the right eye showed absent red fundus reflex, vitreous cells and debris, chorioretinitis and vitritis. Dilated fundus exam of the left eye showed two tiny cotton wool spots along the superior and inferior arcades.

The patient was diagnosed with endogenous endophthalmitis of the right eye as a complication of the systemic ORSA bacteremia, for which he underwent an anterior chamber tap and intravitreal injection of vancomycin, ceftazidime, and voriconazole in the right eye. Prednisolone (1 drop every three hours), and ofloxacin (one drop four times daily) were initiated.

Antibiotics were continued including intravenous vancomycin, gentamycin, levofloxacin, and topical ofloxacin drops. The patient continued to be afebrile and white blood cell count decreased from 18.6 to 7.4.

Later, the anterior chamber fluid aerobic, anaerobic, and fungal cultures came back all negative.

**Figure 3:**
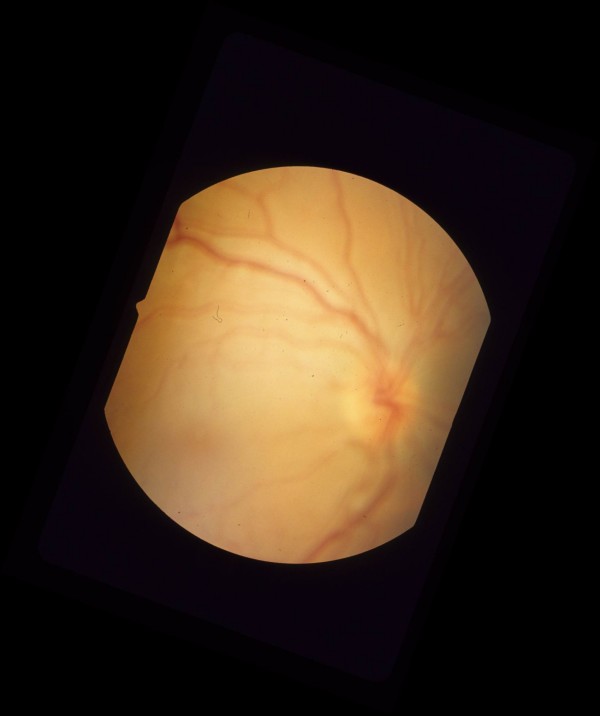
Pre-operative color fundus photograph of the right eye is showing macula-off rhegmatogenous retinal detachment due to a necrotic retinal hole from a flat choroidal lesion inferior to the inferotemporal arcade.

**Figure 4:**
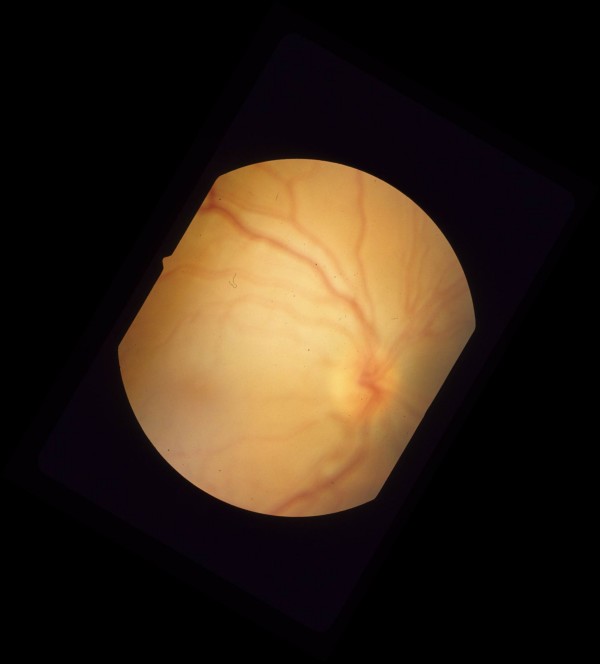
Pre-operative color fundus photograph of the right eye is showing macula-off rhegmatogenous retinal detachment due to a necrotic retinal hole from a flat choroidal lesion inferior to the inferotemporal arcade.

**Figure 5:**
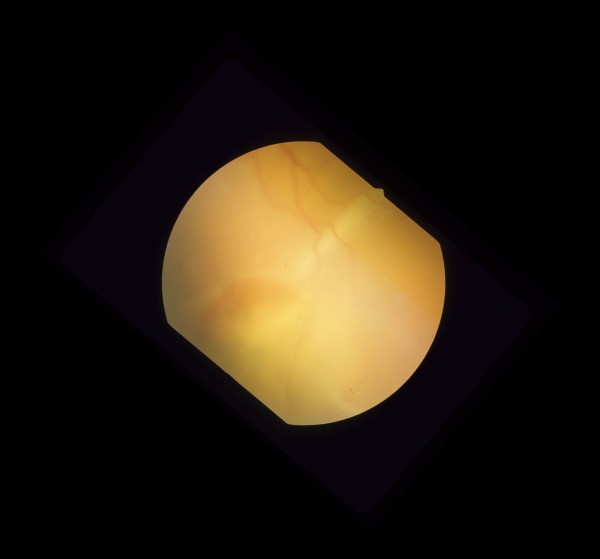
Pre-operative color fundus photograph of the right eye is showing inferotemporal retinal hole and detachment.

Five days post injection exam showed visual acuity of 20/200 on the right, and 20/25 on the left. Intraocular pressure was 8 mmHg on the right, and 10 mmHg on the left. Dilated fundus exam showed decreased chorioretinitis and vitritis in the right eye. However the patient was noted to have macula-off rhegmatogenous retinal detachment, and a retinal hole inferior to the inferotemporal arcade (Figure 3, 4, 5). Dilated fundus exam of the left eye showed decrease in size of the cotton wool spots on the superior and inferior arcades.

The patient was taken to the operating room to repair the retinal detachment. Encircling scleral buckling was performed. Inspection of the posterior pole revealed a macula-off rhegmatogenous detachment and a necrotic retinal hole inferior to the inferotemporal arcade. A posterior vitreous detachment was created by using the vitrector. Following this, the cryoretinopexy was applied to the superotemporal retina where there were some small atrophic holes near the buckle. The peripheral vitreous was trimmed using a scleral depression for 360 degrees without complications. Next, perfluorocarbon was placed on the macula to stabilize the macula, followed by endolaser photocoagulation to surround the retinal hole and also 360 degrees on the scleral buckle. Next, an air-fluid exchange was performed, followed by silicone oil placement. At the end of the operation there was good red reflex and the eye was normotensive by finger palpation.

The patient was continued on intravenous vancomycin and oral levofloxacin for systemic infection control, and was started on one drop of Maxitrol three times daily. Patient was advised to keep his head in a face-down position.

**Figure 6:**
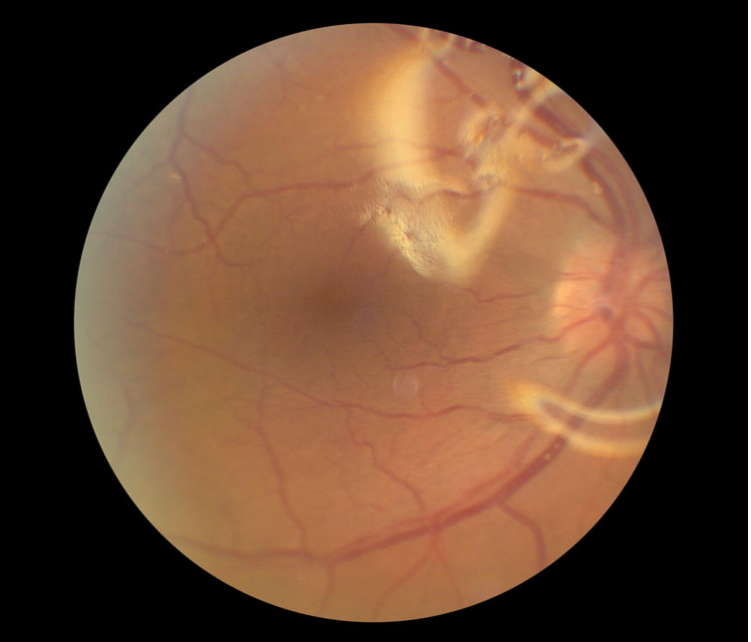
Dilated fundus exam of the right eye three weeks post retinal detachment repair surgery is showing re-attached macula under silicone oil, and flat retina 360 degrees.

**Figure 7:**
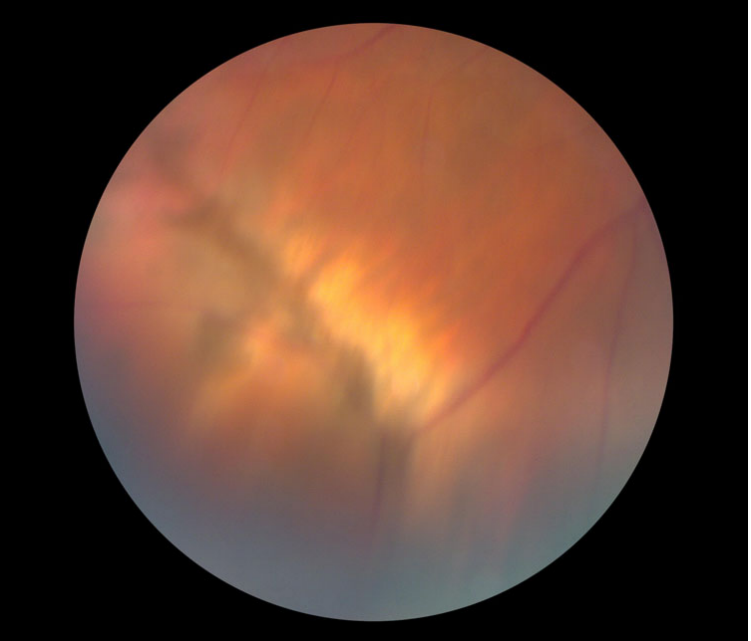
Post-operative color fundus photograph of the right eye is showing treated retinal hole, with laser marking.

Postoperative exam of the right eye showed flat retina 360 degrees (figure 6, 7). The patient was discharged to jail with a plan to continue on intravenous vancomycin, oral levofloxacin, Maxitrol drops, Ofloxacin ophthalmic solution, Prednisolone eye drop, Atropine sulfate ophthalmic solution 1% four times daily, and Cosopt one drop twice daily.

Follow-up exam 4 weeks post operation showed a visual acuity of 20/400 on the right and 20/25 on the left. Slit lamp exam of the right showed deep heme, 1+ cell, no signs of iris neovascularization, and pigmented anterior capsule. Slit lamp Exam of the left eye was normal. Dilated fundus exam of the right eye showed flat retina 360 degrees, bare RPE with surrounding laser scars. Dilated fundus exam of the left eye was normal.

Maxitrol and Ofloxacin were discontinued, atropine dose was reduced to once daily, and patient was given a slow taper down of prednisolone eye drops over 3 weeks. Patient was advised again about the importance of face-down positioning.

Later, the patient was released from jail, and he stopped following with our clinic.

## Discussion

Rhegmatogenous retinal detachment occurs when retinal break allows liquid vitreous access to subretinal space. Incidence is about 1 in 10,000/year; retinal breaks can be found in 97% of cases. Most tears are located superiorly between 10 and 2 o'clock positions. Risk factors are age, history of retinal detachment in the fellow eye (15%), high myopia/axial length (7%), family history, lattice degeneration, trauma, cataract surgery, diabetes, and Nd:YAG laser posterior capsulotomy [9].

Rhegmatogenous RD surgical treatment is achieved by encircling scleral buckling, cryoretinopexy, vitrectomy, endolaser photocoagulation, air-fluid exchange, and silicone oil placement.

In cases where inert gas or sterile air is injected into the vitreous cavity; strict positioning of the patient's head allows gas bubble to contact retinal break and form barrier (RPE pumps subretinal fluid into choroid, allowing retina to reattach), break is sealed with cryo at time of gas injection or sealed with laser once subretinal fluid has resorbed [9].

Complications of cryotherapy are proliferative vitreoretinopathy (PVR), uveitis, cystoid macular edema, intraocular hemorrhage, and chorioretinal necrosis.

Complications of scleral buckle surgery are ischemia (anterior and posterior segments), infection, perforation, strabismus, erosion or extrusion of explant, change in refractive error (induced myopia from increased axial length), macular pucker, cataract, glaucoma, new retinal tears (1.6%), PVR (4%), and failure (5%–10%) [9].

Silicone oil has specific gravity less than water, and surface tension less than that of all gases; therefore, iridectomy is usually made inferiorly; Complications are cataract (100%), band keratopathy (24%), glaucoma (19%), and corneal decompensation (8%) [9].

Scleral buckle has 91% success rate. Final vision depends on macular involvement. Prognosis is worse if macula is detached. Timing of macular detachment is also important, if less than 1 week, 75% of patients recover better than 20/70 visual acuity, if more than 1 week, 50% of patients recover better than 20/70 [9].

Rhegmatogenous retinal detachment can be complicated with PVR where retinal breaks allow cells (Retinal pigment epithelium, glial, myofibroblasts) to proliferate on inner and outer surfaces of retina along scaffold of detached vitreous. Membranes will contract, causing fixed fold and tractional retinal detachment. PVR is the most common reason for failure of retinal reattachment surgery; usually occurs 4 to 6 weeks after the initial repair [9].

## Conclusion

Our case illustrates an interesting presentation of ORSA bacteremia after a lower extremity dog bite, where endogenous endophthalmitis and rhegmatogenous retinal detachment were part of the manifestations. Early diagnosis and intervention are crucial to preserve the visual acuity.

## Competing interests

The authors declare that they have no competing interests.

## Authors' contributions

TA analyzed and interpreted the patient data, and wrote the manuscript. SE helped with collecting the data, and was a contributor in writing the manuscript. RS was the retina fellow who performed the intravitreal injection and the retinal detachment repair surgery. DE was the retina attending who staffed the retinal detachment repair surgery. All authors read and approved the final manuscript.

## Consent

Written informed consent was obtained from the patient for publication of this case report and accompanying images. A copy of the written consent is available for review by the Editor-in-Chief of this journal.
